# Exploring the Relationship between MicroRNAs, Intratumoral Microbiota, and Breast Cancer Progression in Patients with and without Metastasis

**DOI:** 10.3390/ijms25137091

**Published:** 2024-06-28

**Authors:** Aurora Laborda-Illanes, Lucía Aranega-Martín, Lidia Sánchez-Alcoholado, Soukaina Boutriq, Isaac Plaza-Andrades, Jesús Peralta-Linero, Guadalupe Garrido Ruiz, Bella Pajares-Hachero, Martina Álvarez, Emilio Alba, Alicia González-González, María Isabel Queipo-Ortuño

**Affiliations:** 1 Clinical Management Unit of Medical Oncology, Regional and Virgen de la Victoria University Hospitals-IBIMA BIONAND-CIMES-UMA Platform, 29010 Málaga, Spain; aurora.laborda@ibima.eu (A.L.-I.); lucia.aramar97@uma.es (L.A.-M.); lsalcoholado@gmail.com (L.S.-A.); soukaina@ibima.eu (S.B.); isaac.plaza.andrades@ibima.eu (I.P.-A.); jesus.peralta@ibima.eu (J.P.-L.); bella.pajares@ibima.eu (B.P.-H.); martina@uma.es (M.Á.); ealbac@uma.es (E.A.); 2 Faculty of Medicine, University of Málaga, Andalucía Tech, Campus de Teatinos s/n, 29071 Málaga, Spain; 3 UGC Radiodiagnosis, Virgen de la Victoria University Hospital, 29010 Málaga, Spain; gugarrid@gmail.com; 4 Department of Human Physiology, Human Histology, Pathological Anatomy and Physical Education, Faculty of Medicine, University of Málaga, 29071 Málaga, Spain; 5 Department of Medicine, Faculty of Medicine, University of Málaga, 29071 Málaga, Spain; 6 UGC Endocrinology and Nutrition, Regional University Hospital of Málaga, Institute of Biomedical Research of Málaga (IBIMA), Faculty of Medicine, University of Málaga, 29071 Málaga, Spain; 7 Department of Surgical Specialties, Biochemistry and Immunology, Faculty of Medicine, University of Málaga, 29071 Málaga, Spain

**Keywords:** microRNAs, microbiota, breast cancer, metastasis, prognostic signature

## Abstract

Breast cancer (BC) continues to pose a significant burden on global cancer-related morbidity and mortality, primarily driven by metastasis. However, the combined influence of microRNAs (miRNAs) and intratumoral microbiota on BC metastasis remains largely unexplored. In this study, we aimed to elucidate the interplay between intratumoral microbiota composition, miRNA expression profiles, and their collective influence on metastasis development in BC patients by employing 16S rRNA sequencing and qPCR methodologies. Our findings revealed an increase in the expression of miR-149-5p, miR-20b-5p, and miR-342-5p in metastatic breast cancer (Met-BC) patients. The Met-BC patients exhibited heightened microbial richness and diversity, primarily attributed to diverse pathogenic bacteria. Taxonomic analysis identified several pathogenic and pro-inflammatory species enriched in Met-BC, contrasting with non-metastatic breast cancer (NonMet-BC) patients, which displayed an enrichment in potential probiotic and anti-inflammatory species. Notably, we identified and verified a baseline prognostic signature for metastasis in BC patients, with its clinical relevance further validated by its impact on overall survival. In conclusion, the observed disparities in miRNA expression and species-level bacterial abundance suggest their involvement in BC progression. The development of a prognostic signature holds promise for metastasis risk assessment, paving the way for personalized interventions and improved clinical outcomes in BC patients.

## 1. Introduction

Breast cancer (BC) is a widespread malignancy among women worldwide and the leading cause of cancer-related deaths [1,2]. BC exhibits remarkable heterogeneity, encompassing multiple subtypes with distinct clinical outcomes [3]. Nevertheless, despite significant advancements in surgical and postsurgical treatments, nearly 20% of early-stage BC patients develop metastasis [4,5].

MicroRNAs (miRNAs) have emerged as key players in cancer research due to their pivotal roles in various aspects of cancer biology, including tumorigenesis, invasion, metastasis, relapse, and drug resistance [5,6]. Extensive research using in vitro and in vivo models has demonstrated how aberrant miRNA expression can modulate signaling pathways involved in various cancers, including breast, lung, and colon cancer, among others [3,7]. Moreover, miRNAs can act as competitive onco-miRs and tumor suppressor miRNAs, with their net effect determined by the intricate balance of their expression levels within signaling pathways, interactions with other tumor-related factors, and their impact on the immune system’s response to cancer [8]. In fact, miRNAs can play a role in altering the energy metabolism of tumor cells, which in turn affects the composition of the tumor microenvironment, making it immunosuppressive and facilitating immune evasion and metastatic progression [5,9].

On the other hand, recent research has shed light on the role of intratumoral microbiota in BC metastasis. Intratumoral microbiota are bacteria that reside within tumors and can affect tumor growth and progression through various mechanisms, such as modulating the immune response, altering cell signaling pathways, and influencing the tumor microenvironment [10,11,12,13]. These intratumoral bacteria can travel through the circulation system together with the cancer cells, playing critical roles in metastatic colonization [14]. Studies have shown that depleting breast intratumoral bacteria can significantly reduce lung metastasis in murine breast tumor models [14,15]. The intriguing connection between intratumoral bacteria and cancer progression and metastasis may be linked to miRNA regulation, as bacteria can modulate miRNA expression, influencing downstream pathways and shaping the tumor microenvironment [16]. Butyrate, a bacterial product, has been shown to affect miRNA expression in colorectal cancer cells, impacting cell proliferation, metastasis, and angiogenesis [9,16]. *Fusobacterium nucleatum* and *Escherichia coli* have also been implicated in miRNA-mediated mechanisms that influence cancer progression [17,18]. Nevertheless, this relationship between miRNAs and microbiota appears to be bidirectional, as miRNAs can also influence the survival and composition of bacteria [9]. For example, miRNAs can influence bacterial abundance in the tumor environment by regulating glucose metabolism, thereby modulating tumor growth [16].

In our study, we aimed to uncover the potential link between tumoral miRNA expression, intratumoral microbiota, and the presence of metastasis in BC patients. To achieve this, we investigated the differential tumoral expression of specific miRNAs, including miR-149-5p, miR-10a-5p, miR-20b-5p, miR-30a-3p, and miR-342-5p, along with the composition of intratumoral microbiota through 16S RNAr sequencing in BC patients with and without metastasis. The five miRNAs chosen for this study were identified from a previous study that validated a multi-miRNA-based model from a total of 1105 different miRNAs. In this previous study, we observed significant expression differences in these selected miRNAs between BC patients with early metastasis and those who remained disease-free 5 years post-surgery [19]. While the association between miRNAs and microbiota has been explored in other cancer types, this study represents a novel exploration of this interplay in BC.

## 2. Results

### 2.1. Baseline Characteristics of the Study Patients

Table 1 summarizes the clinical characteristics of the study patients. The median age at diagnosis was 60 (range, 31–85) years. The patients were grouped based on the age at diagnosis as younger or older than 50 years in the NonMet-BC and Met-BC groups.

Concerning the hormonal status, we had 12 (28.6%) and 19 (25.7%) premenopausal patients and 30 (71.4%) and 55 (74.3%) postmenopausal patients in the NonMet-BC and Met-BC groups, respectively. Intrinsic subtypes were grouped in the NonMet-BC and Met-BC groups, respectively, as follows: 7 (16.7%) and 16 (21.6%) were identified as Luminal A, 20 (47.6%) and 26 (35.1%) as Luminal B-HER2 negative, 6 (14.3%) and 4 (5.4%) as Luminal B-HER2 positive, 5 (14.3%) and 15 (20.3%) as triple-negative tumors, and 3 (7.1%) and 13 (17.6%) as HER2 enriched. Related to the location of the first metastasis process, the most frequent sites were bone (28.4%), liver (24.3%), and lung (13.5%).

### 2.2. Differential microRNA Expression in Breast Tumor Tissue of Breast Cancer Patients with and without Metastasis

**Figure 1:**
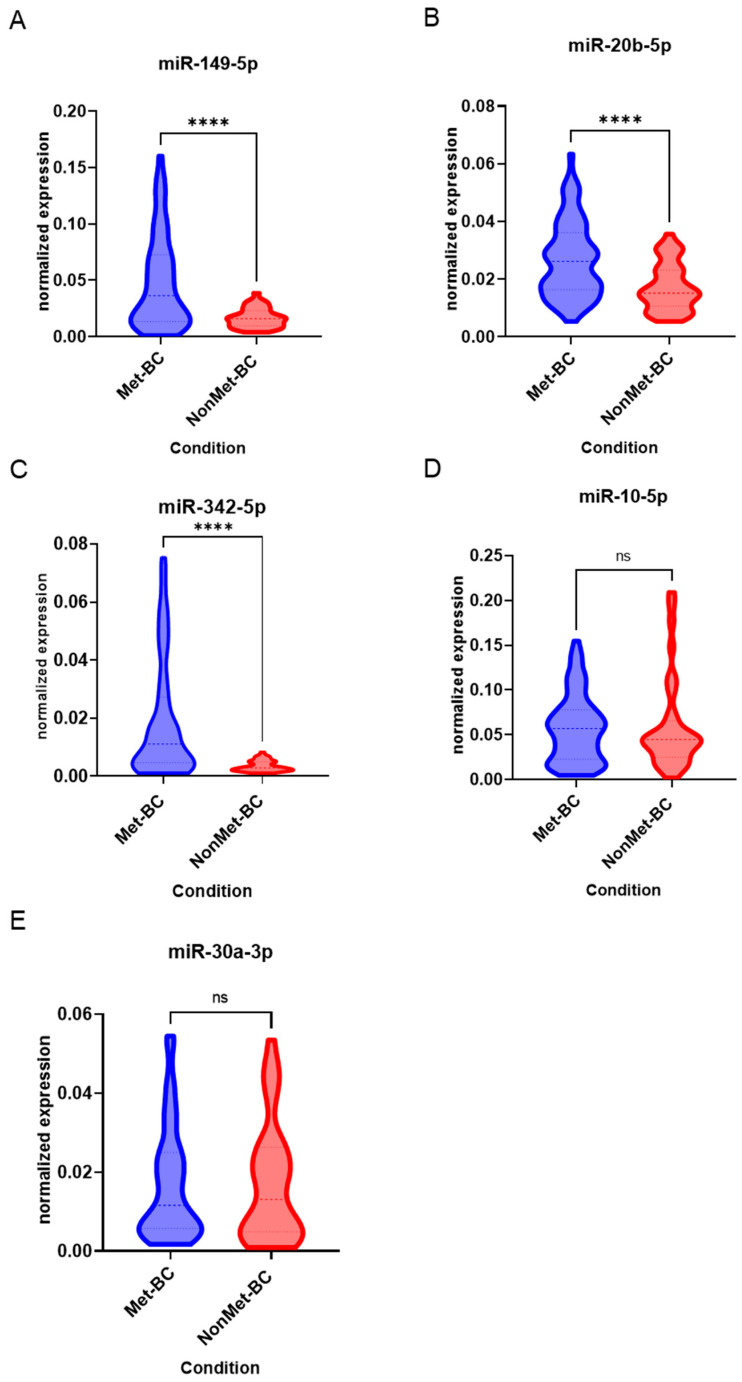
Violin plots depicting the normalized expression levels of the examined miRNAs, revealing significant distinctions between patients with metastatic breast cancer (Met-BC) and those with non-metastatic breast cancer (NonMet-BC). The panels display the expression profiles of (**A**) miR-149-5p, (**B**) miR-20b-5p, (**C**) miR-342-5p, (**D**) miR-10a-5p, and (**E**) miR-30a-3p. (**** *p* < 0.0001; ns = not significant).

The analysis of microRNA (miRNA) expression, including miR-149-5p, miR-20b-5p, miR-342-5p, miR-10a-5p, and miR-30a-3p, in tumor samples revealed significant differences between Met-BC and NonMet-BC patients. Notably, miR-149-5p, miR-20b-5p, and miR-342-5p exhibited higher expression levels in the Met-BC group compared to the non-Met-BC group (*p* < 0.001) (Figure 1A–C). However, miR-10a-5p and miR-30a-3p levels did not display significant differences between the two study groups (Figure 1D,E).

### 2.3. Differences in Taxonomic Composition of Intratumoral Bacteria in Breast Cancer Patients between Metastatic and Non-metastatic Clinical State

**Figure 2:**
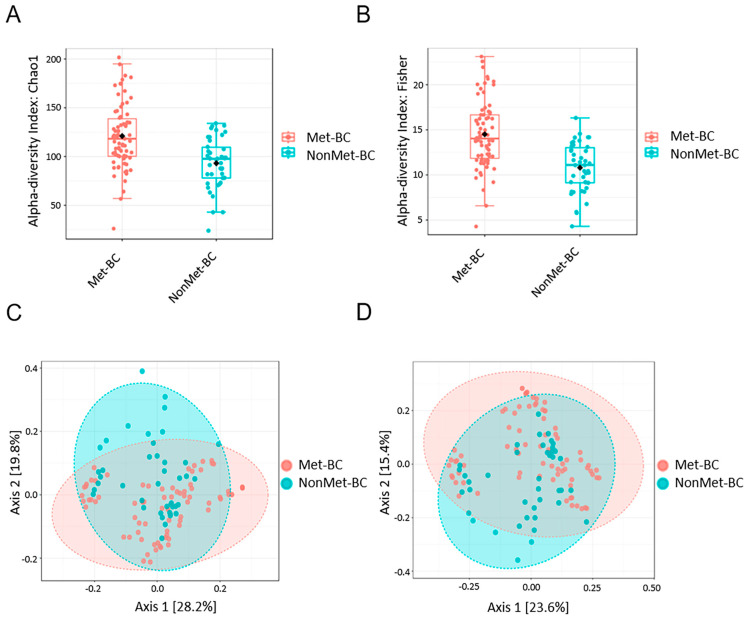
Comparison of alpha and beta diversity between the study groups. (**A**) Chao1 index; (**B**) Fisher index; (**C**,**D**) Principal component plot based on the Bray–Curtis distance matrix and the Jaccard indices from the metastatic breast cancer (Met-BC) and those with non-metastatic breast cancer (NonMet-BC) patients at genus level. The first two coordinates are plotted with the percentage of variability, which is explained and indicated on the axis.

Alpha diversity was evaluated at the genus level using the Chao1 (community richness) and Fisher (microbiota diversity) indices. The Chao1 and Fisher values for both groups exhibited a significant increase in richness and diversity within the Met-BC group compared to the NonMet-BC group (Chao1 *p* < 0.001; Fisher *p* < 0.001). Thus, the microbiota in the Met-BC group presented higher bacterial taxa and genera evenness (Figure 2A,B).

Furthermore, to assess beta diversity analysis between the two groups, we used the Bray–Curtis dissimilarity and Jaccard indices. The cluster plots (PCoA) displayed a notable separation in bacterial communities between the Met-BC and NonMet-BC groups (Bray–Curtis index *p*-value = 0.001, PERMANOVA) and (Jaccard index *p*-value = 0.001, PERMANOVA), indicating a significant difference in the distribution and variability in the microbiota profile between the groups (Figure 2C,D).

**Figure 3:**
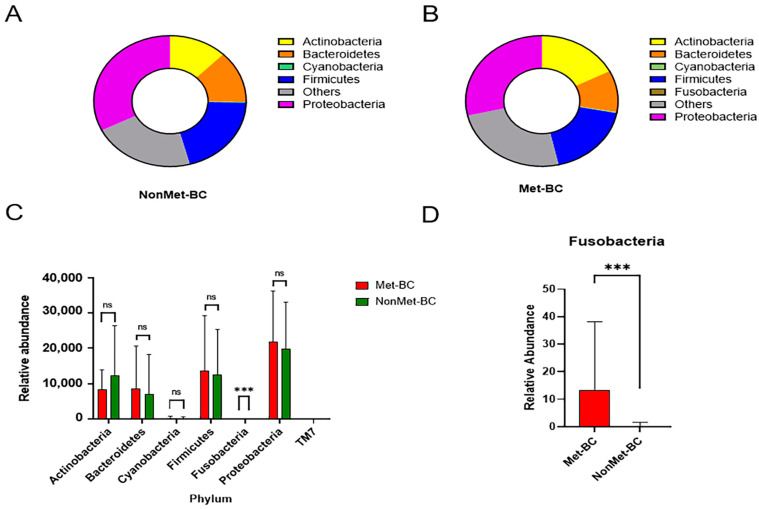
Taxonomic composition of the intratumoral breast microbiota depicted as average relative abundances at the phylum, genus, and species level in both groups. (**A**,**B**) Non-metastatic breast cancer (NonMet-BC) group and metastatic breast cancer (Met-BC) group at phylum level; (**C**) Relative abundance of the different phyla in the Met-BC and NonMet-BC groups; (**D**) Differential abundance of the phylum *Fusobacteria* between the Met-BC and NonMet-BC groups (*** *q* < 0.001; ns = not significant).

On the other hand, the analysis focused on the composition of intratumoral BC microbiota at the phylum level, revealing a noteworthy disparity solely in the abundance of Fusobacteria (*q* < 0.001) between the two study groups. Notably, the Fusobacteria levels exhibited a pronounced increase in the Met-BC group (Figure 3A–D).

**Figure 4:**
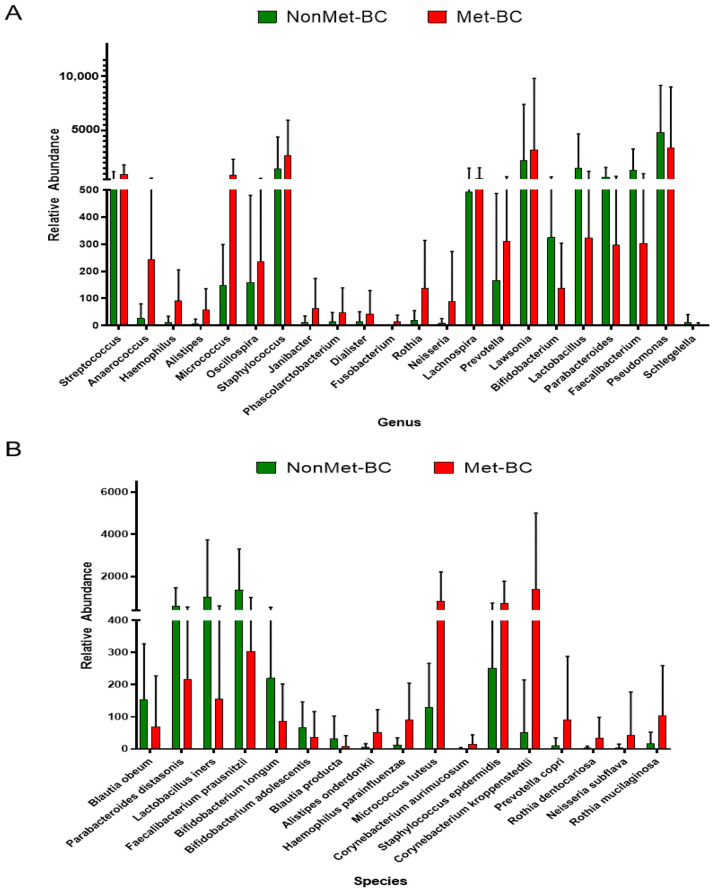
Significant differential bacteria of the intratumoral microbiota composition (*q* < 0.05) at the genus level (**A**) and species (**B**) levels between the Met-BC and NonMet-BC groups by (*p* < 0.05).

At the genus level, we observed significant differences in microbial composition between the Met-BC group and the NonMet-BC group. A significant increase was found in the following genera in the Met-BC group in comparison with the NonMet-BC group: *Streptococcus* (*q* < 0.001), *Anaerococcus* (*q* < 0.001), *Haemophilus* (*q* < 0.001), *Alistipes* (*q* < 0.001), *Micrococcus* (*q* < 0.001), *Oscillospira* (*q* < 0.001), *Staphylococcus* (*q* < 0.001), *Janibacter* (*q* = 0.002), *Phascolarctobacterium* (*q* = 0.006), *Dialister* (*q* = 0.002), *Fusobacterium* (*q* = 0.002), *Rothia* (*q* = 0.003), *Neisseria* (*q* = 0.002), *Lachnospira* (*q* = 0.004), *Prevotella* (*q* < 0.001), and *Lawsonia* (*q* = 0.007). In contrast, we found a significant increase at the genus level in the NonMet-BC group in *Bifidobacterium* (*q* < 0.001), *Lactobacillus* (*q* < 0.001), *Parabacteroides* (*q* = 0.002), *Faecalibacterium* (*q* < 0.001), *Pseudomonas* (*q* = 0.006), and *Schlegelella* (*q* = 0.010) (Figure 4A).

At the species level, we identified 17 species with significant differences in abundance between the Met-BC and NonMet-BC groups. Specifically, there was a significant increase in the abundance of *Blautia obeum* (*q* < 0.001), *Parabacteroides distasonis* (*q* < 0.001), *Lactobacillus iners* (*q* = 0.003), *Faecalibacterium prausnitzii* (*q* < 0.001), *Bifidobacterium longum* (*q* = 0.01), *Bifidobacterium adolescentis* (*q* = 0.008), and *Blautia producta* (*q* = 0.008) in the NonMet-BC group compared to the Met-BC group. Conversely, in the Met-BC group, we observed an increase in the abundance of *Alistipes onderdonkii* (*q* < 0.001), *Haemophilus parainfluenzae* (*q* < 0.001), *Micrococcus luteus* (*q* < 0.001), *Corynebacterium aurimucosum* (*q* < 0.001), *Staphylococcus epidermidis* (*q* < 0.001), *Corynebacterium kroppenstedtii* (*q* < 0.001), *Prevotella copri* (*q* < 0.001), *Rothia dentocariosa* (*q* = 0.008), *Neisseria subflava* (*q* = 0.015), and *Rothia mucilaginosa* (*q* = 0.004) (Figure 4B).

**Figure 5:**
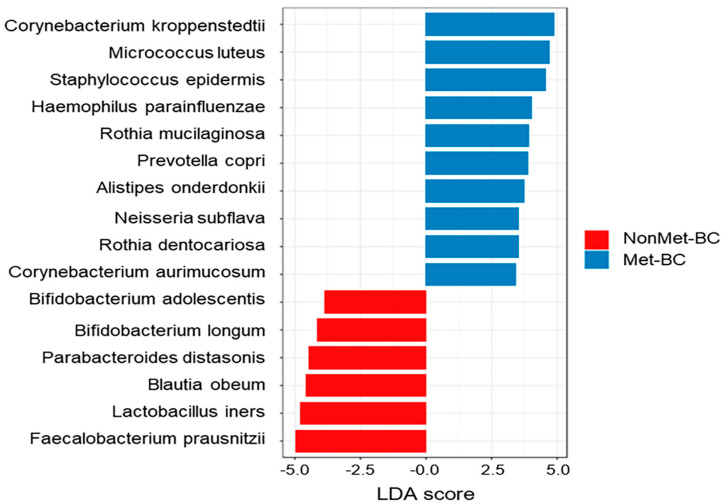
The LDA effect size (LEfSe) based on the 16S rRNA gene sequencing between the metastatic breast cancer (Met-BC) and non-metastatic breast cancer (NonMet-BC) groups exhibited the biomarker bacteria of intratumoral microbiota at the species level that characterizes the significant differences between the two groups (LDA > 3, *q* < 0.05).

Finally, we also employed LEfSe analysis to identify intratumoral microbiota biomarkers associated with metastasis in BC patients. These findings underscore metastasis-specific alterations in the intratumoral microbiota in the context of metastatic BC. At the species level, we identified significant differences between the two study groups. Of these discriminatory taxa, *Corynebacterium kroppenstedtii*, *Corynebacterium aurimucosum*, *Rothia mucilaginosa, Rothia dentocariosa*, and *Alistipes onderdonkii* were found to be significantly abundant in the Met-BC group, whereas 6 species, some of which had probiotic and anti-inflammatory activity, such as *Bifidobacterium adolescentis*, *Bifidobacterium longum*, *Lactobacillus iners*, and *Faecalibacterium prausnitzii*, were significantly enriched in the NonMet-BC group (Figure 5).

### 2.4. Differences in Intratumoral Microbiota Functions and the Associations between Key Pathways and Bacterial Species in Metastatic and Non-Metastatic Breast Cancer Patients

To assess the functional potential of the microbial communities, we conducted an analysis of the predicted metagenomes based on 16S rRNA sequencing data using the PICRUSt2 algorithm. Our results offer a comprehensive overview of the functional diversity and potential metabolic pathways within intratumoral BC.

Notably, we identified 33 pathways exhibiting significantly greater representation in the Met-BC group than in the NonMet-BC group. Among these, several pathways deserve special mention, encompassing processes such as bacterial invasion (bacterial invasion of epithelial cells), enhanced transcription (basal transcription factors), inter- and intracellular communication (lysosome), cell signaling (Wnt signaling pathway, Notch signaling pathway), extracellular matrix remodeling (glycosaminoglycan degradation, other glycan degradation), biosynthesis of various biological compounds (N-glycan biosynthesis, glycosphingolipid biosynthesis—ganglio series, globo series, and lacto and neolacto series), protein degradation involving tumor suppressor proteins (proteasome), xenobiotics metabolism (cytochrome P450), and cancer-related pathways (bladder cancer).

**Figure 6:**
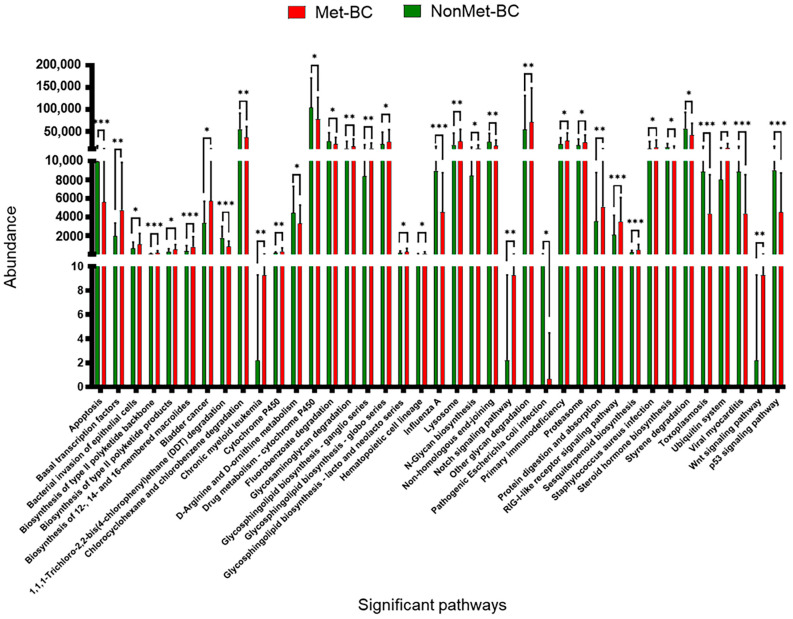
Enrichment of different KEGG pathways showing significant differences between the metastatic breast cancer (Met-BC) and non-metastatic breast cancer (NonMet-BC) groups calculated using two-sided unpaired Mann–Whitney test (*p* < 0.05) (* *p* < 0.05; ** *p* < 0.01; *** *p* < 0.001.).

Conversely, the NonMet-BC group exhibited significantly enriched pathways, such as apoptosis, xenobiotic biodegradation, and metabolism (drug metabolism—cytochrome P450, fluorobenzoate degradation, 1,1,1-Trichloro-2,2-bis(4-chlorophenyl)ethane (DDT) degradation, chlorocyclohexane and chlorobenzene degradation, and styrene degradation), pathways related to bacterial and viral infections (influenza A, pathogenic *E. coli* infection, toxoplasmosis, viral myocarditis), non-homologous end-joining, and the tumor suppressor p53 signaling pathways (Figure 6).

Our analysis explored the correlation between the key pathways and the bacterial species, showing significant differences at the species level between the Met-BC and NonMet BC groups. Among the pathways increased in the Met-BC group, the bacterial invasion of epithelial cells and Notch signaling pathway exhibited a significant positive association with the presence of *Staphylococcus epidermidis* (r = 0.823, *p* < 0.001; r = 0.322, *p* < 0.001) and *Corynebacterium kroppenstedtii* (r = 0.525, *p* < 0.001; r = 0.352, *p* < 0.001). In contrast, pathways enriched in the NonMet-BC group displayed noteworthy positive correlations, between *Parabacteroides distasonis* and *Faecalibacterium prausnitzii* with 1,1,1-Trichloro-2,2-bis(4-chlorophenyl)ethane (DDT) degradation (r = 0.415, *p* < 0.001 and r = 0.445, *p* < 0.001, respectively), *Bifidobacterium longum*, and *Faecalibacterium prausnitzii* with drug metabolism—cytochrome P450 (r = 0.350, *p* < 0.001; r = 0.323, *p* < 0.001) and apoptosis (r = 0.260, *p* < 0.01; r = 0.212, *p* < 0.05, respectively).

### 2.5. Relationship between Tumor Breast Tissue Microbiota and microRNA Expression Levels in Metastatic and Non-metastatic Breast Cancer Patients

We conducted correlation analyses to assess the associations between bacterial species with differential abundance and miRNA expression in BC tumor tissues of both study groups.

Seven bacteria, Alistipes onderdonkii (r = 0.214; *p* = 0.021), Corynebacterium kroppenstedtii (r = 0.244; *p* = 0.008), Haemophilus parainfluenciae (r = 0.217; *p* = 0.020), Micrococcus luteus (r = 0.226; *p* = 0.015), Neisseria subflava (r = 0.266; *p* = 0.004), Prevotella copri (r = 0.198; *p* = 0.033), and Staphylococcus epidermis (r = 0.197; *p* = 0.034), which were most abundant in the Met-BC group, presented positive associations with miR-149-5p. In contrast, miR-149-5p displayed a negative association with Blautia obeum (r = −0.231; *p* = 0.013), Faecalibacterium prausnitzii (r = −0.242; *p* = 0.009), and Parabacteroides distasonis (r = −0.278; *p* = 0.003), which were found at a higher abundance in the non-Met-BC group. For miR-20b-5p, positive correlations were also observed with Corynebacterium kroppenstedtii (r = 0.239; *p* = 0.010), Haemophilus parainfluenzae (*p* = 0.038; r = 0.193), and Prevotella copri (r = 0.225; *p* = 0.015), while negative correlations were noted with Blautia obeum (r = −0.200; *p* = 0.031) and Parabacteroides distasonis (r = −0.239; *p* = 0.010).

**Figure 7:**
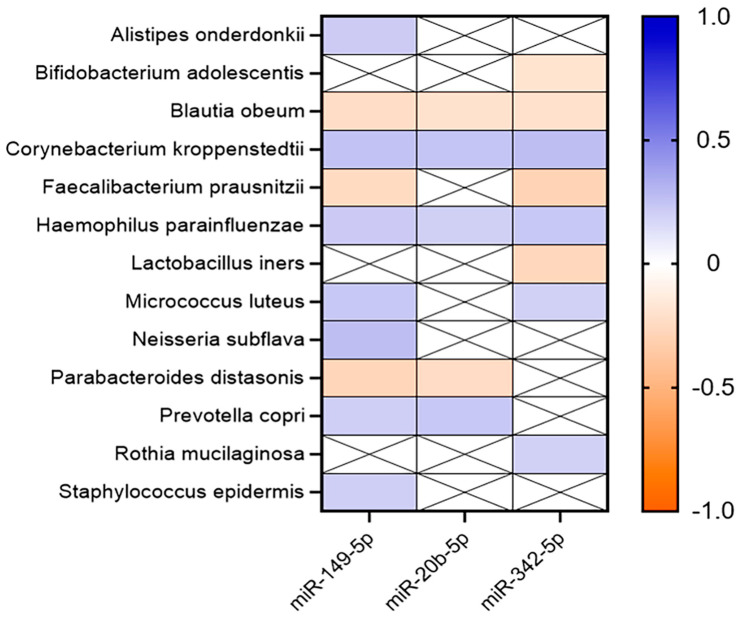
Heatmap representing the correlation analysis among significant miRNAs and Met-BC and NonMet-BC breast cancer microbiota at species level (*p* < 0.05). X: no significant correlation.

Lastly, miR-342-5p showed positive correlations with *Corynebacterium kroppenstedtii* (r = 0.266; *p* = 0.004), *Haemophilus parainfluenzae* (*p* = 0.015; r = 0.226), *Micrococcus luteus* (r = 0.190; *p* = 0.042), and *Rothia mucilaginosa* (r = 0.190; *p* = 0.041), and negative correlations with *Bifidobacterium adolescentis* (r = −0.187; *p* = 0.044), *Blautia obeum* (r = −0.207; *p* = 0.026), *Faecalibacterium prausnitzii* (r = −0.290; *p* = 0.002), and *Lactobacillus iners* (r = −0.265; *p* = 0.004) (Figure 7).

### 2.6. Baseline Intratumoral Microbiota and microRNAs Could Predict Metastasis Development in Breast Cancer Patients and Are Associate with Overall Survival

Having outlined the notable distinctions in both the composition of the intratumoral microbiota and miRNA expression between the NonMet-BC and Met-BC groups, we subsequently assessed the prognostic power of intratumoral microbiota and miRNAs in relation to metastasis development in BC patients. An RF algorithm was employed to construct a prognostic model. This model was based on the overall intratumoral microbial profiles and miRNAs, utilizing the species-level relative abundance data and the relative miRNA expressions as inputs. Multiple RF analyses were generated in the training phase to identify the most effective model for metastasis prediction in BC patients, and the best-performing model was selected as the final model.

**Figure 8:**
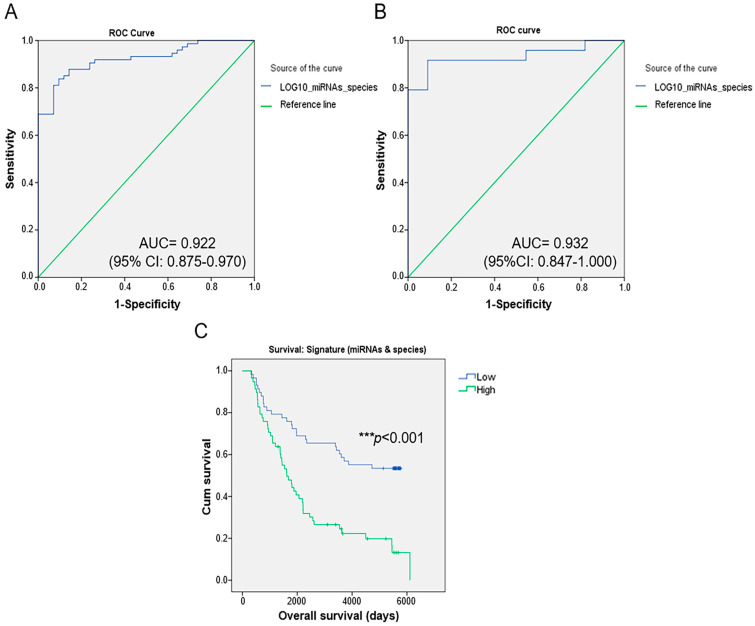
Random forest (RF) analysis to distinguish between breast cancer patients who develop metastases based on the abundance of intratumoral microbiota and miRNA expression. (**A**) Receiver operating characteristic (ROC) curve and area under the curve (AUC) were generated to predict the presence of metastasis in the training cohort based on the combined signature of the five intratumoral species and the two miRNAs (miR-149-5p, miR-342-5p, *Corynebacterium kroppenstedtii*, *Haemophilus parainfluenzae*, *Alistipes onderdonkii*, *Blautia obeum*, and *Corynebacterium aurimucosum*) selected using RF (AUC value 0.922; 95% CI: 0.875–0.970); (**B**) ROC curve was generated to validate the signature for predicting metastasis in the validation cohort (AUC value 0.932; 95% CI: 0.847–1.000); (**C**) Kaplan–Meier analysis to estimate the overall survival of breast cancer patients with and without metastasis in the training cohort based on the selected signature (*** *p* < 0.001).

This model selected five bacterial species together with two miRNAS as the most important features and had a robust and statistically significant diagnostic accuracy, with an AUC of 0.922 (95% CI: 0.875–0.970) (Figure 8A). The bacterial species and miRNAs accounting for this model were *Alistipes onderdonkii*, *Corynebacterium kroppenstedtii*, *Haemophilus parainfluenzae*, *Blautia obeum*, and *Corynebacterium aurimucosum* and miR-342-5p and miR-149-5p. From the five species and two miRNAs selected by the optimized model, four species and two miRNAs were significantly increased in the Met-BC patients compared to the NonMet-BC group. The RF model with the same parameters was used for metastasis prediction on a validation cohort consisting of 35 BC patients (24 with metastasis and 11 without metastasis). After an RF analysis in this validation cohort, the AUC value of the selected model was 0.932 (95%CI: 0.847–1.000) (Figure 8B).

Kaplan–Meier analyses were performed to examine the association between this model and patient overall survival (OS). The patients were divided into high and low values of the signature integrating miRNA expression and species abundance, using the median as the cutoff point. Compared with the low-value group, patients in the high-value group had significantly (*p* < 0.001) shorter OS (Figure 8C).

## 3. Discussion

The findings presented in this pilot study bring forth a wealth of knowledge regarding the interplay of intratumoral microbiota, miRNAs, and metastasis in the context of BC. We have demonstrated the existence of a significant association between specific intratumoral microbiota taxa and several onco-miRNAs for metastasis development in BC patients. Moreover, we have described the prognostic capacity of a signature formed using five intratumoral enriched bacterial species and two onco-miRNAs to test the development of metastasis using well-characterized training and validation cohorts. Whereas *Alistipes onderdonkii*, *Corynebacterium kroppenstedtii*, *Haemophilus parainfluenzae*, *Corynebacterium aurimucosum*, and miR-342-5p and miR-149-5p were overrepresented in Met-BC patients and chosen as discriminatory variables in our metastasis-prediction RF model, *Blautia obeum* was overrepresented in the NonMet-BC patients. In addition, Kaplan–Meier analysis proved that patients with a low value of this signature had a significantly higher OS than those with a high value, suggesting that the baseline signature might also predict the OS of patients with BC.

In this study, we observed an increase in the richness and diversity of the intratumoral microbiota in the Met-BC patients, which raises intriguing questions about the role of intratumoral microbial communities in BC progression. This diversity could potentially be indicative of a more complex and dynamic microenvironment in metastatic tumors. The significant differences in bacterial taxa abundance identified at the genus and species levels between the Met-BC and NonMet-BC patients are relevant. In fact, *Corynebacterium kroppenstedtii*, *Corynebacterium aurimucosum*, *Rothia mucilaginosa*, *Rothia dentocariosa*, *Haemophilus parainfluenzae*, and *Staphylococcus epidermis* were enriched in the Met-BC compared to NonMet-BC patients. These data are consistent with previous research linking several of these bacteria to inflammation and cancer proliferation [20,21,22,23]. *Corynebacterium kroppenstedtii* has been associated with granulomatous mastitis and breast abscess [24,25], while *Corynebacterium aurimucosum* has been implicated in non-responsiveness to anti-PD1 treatment in epithelial tumors [26]. On the other hand, *Staphylococcus epidermis* is believed to exhibit significant inflammatory activity and induce elevated levels of regulatory T cells [27]. The *Rothia* genus, encompassing species such as *Rothia mucilaginosa* and *Rothia dentocariosa*, has been found to be elevated in patients with BC and, notably, in patients with metastasis in oral squamous cell carcinoma [28,29]. Finally, *Haemophilus influenzae* was associated with genes representing crucial pathways for tumorigenesis, such as E2 signaling, G2M checkpoint, and mitotic spindle assembly in BC patients [30].

On the contrary, in the NonMet-BC patients, we observed an increased abundance of specific bacterial species with probiotic and anti-inflammatory activities, such as *Bifidobacterium adolescentis*, *Bifidobacterium longum*, *Lactobacillus iners*, *Parabacteroides distasonis*, *Blautia obeum,* and *Faecalibacterium prausnitzii*, hinting at a possible protective role against metastasis [31,32]. The higher abundance of *Bifidobacterium longum* in the intestinal microbiota has been linked to a favorable response in patients with hormone receptor-positive (HR+) HER2-negative metastatic BC who received cyclin-dependent kinase (CDK)4/6 inhibitors as part of their endocrine therapy [33]. In an in vitro experiment, it was demonstrated that lactic acid, a metabolite produced by *Lactobacillus iners*, triggers the activation of the Wnt pathway via the lactate–Gpr81 complex. This activation subsequently leads to an increase in the level of core fucosylation in epithelial cells, resulting in the inhibition of the proliferation and migration of cervical cancer cells [34]. Koh et al. demonstrated that *Parabacteroides distasonis* mitigates tumorigenesis, modulates inflammatory markers, and enhances intestinal barrier integrity in azoxymethane-treated A/J mice [35]. A higher abundance of *Blautia obeum* was associated with improved progression-free survival in HER2-negative metastatic BC patients receiving capecitabine treatment [36]. Finally, the reduction in the abundance of *Faecalibacterium prausnitzii*, a prominent butyrate-producing gut bacterium, is associated with a decrease in short-chain fatty acids, particularly propionate production, possibly contributing to BC progress [37].

Similar to our study, in patients who developed metastasis in various types of tumors, bacterial pathways related to bacterial invasion of epithelial cells, inter- and intracellular communication, cell signaling, extracellular matrix remodeling, and cancer, among others, were significantly upregulated [38,39,40,41,42]. These pathways might contribute to the metastatic cascade by modulating the tumor microenvironment. Fu et al. discovered that intracellular microbiota plays a critical role in tumor metastasis by influencing the cellular cytoskeleton and cell viability under mechanical stress. Their findings revealed that cancer cells employ intracellular microbiota to survive the fluid shear stress in the circulation during metastatic colonization. This survival advantage of tumor bacteria is most pronounced during metastasis, rather than primary tumor growth. This mechanism extends beyond BC and is also evident in colorectal cancer, where intratumor microbiota persists during metastasis and passages [14]. Moreover, in this study, a positive correlation was observed between *Staphylococcus epidermidis* and *Corynebacterium kroppenstedtii* (enriched in metastatic patients) with the pathway bacterial invasion of epithelial cells (a process wherein bacteria penetrate and enter these cells). Staphylococci express surface proteins such as Fn-binding proteins, aiding adhesion to extracellular matrices or host cells, and invasion of non-professional phagocytic cells (epithelial and endothelial cells). This adhesion is crucial for biofilm formation and host cell invasion, protecting bacteria from the immune system. *Staphylococcus aureus* adheres to cells via the FnBP–Fn-α5β1 integrin pathway, triggering a signaling cascade (FAK, Src, PI3K, Akt). This mobilizes the actin cytoskeleton, enabling entry into host cells and enabling intracellular persistence and chronic infections [43]. Our findings suggest that specific tumor-resident microbiota can have a significant role in promoting BC metastasis and enhancing the survival of cancer cells during tumor progression.

On the other hand, the identification of the overexpression of miR-149-5p, miR-20b-5p, and miR-342-5p in the Met-BC compared to NonMet-BC patients reveals the importance of miRNAs coming from BC tissue as possible diagnostic molecular biomarkers of metastasis. Consistent with our findings, previous research has also reported that the overexpression of miR-20b-5p in human BC tissues and cell lines inhibits the translation of the tumor suppressor PTEN mRNA, thereby enhancing the proliferation, migration, and wound-healing abilities of ZR-75-30, MCF-7, and T47D BC cells, while also suppressing apoptosis [44,45]. However, miR-149-5p and miR-342-5p exhibit conflicting roles in the existing scientific literature, being documented as both onco-miRs and tumor suppressor miRNAs in diverse cancer investigations [46,47,48,49]. Specifically, in the context of triple-negative BC, reduced expression of miR-149-5p has been correlated with enhanced macrophage infiltration and diminished patient survival, mediated through the epidermal growth factor (EGF) pathway [50]. In chemoresistant ovarian cancer tissues, elevated miR-149-5p expression has been consistently reported relative to chemosensitive counterparts, involving the Hippo signaling pathway and resulting in the inactivation of TEAD expression [51]. In recent studies in BC patients, miRNA-342-5p has exhibited the highest expression in ER-positive and HER2+ luminal B tumors, showing a positive correlation between miR-342 expression and ERα expression [52]. In this study, significant positive associations have been established between the expression of miR-149-5p, miR-20b-5p, and miR-342-5p (which possess an onco-mir character or promote metastasis) and species considered pathogenic and associated with poor cancer prognosis, such as *Corynebacterium kroppenstedtii*, *Haemophilus parainfluenzae*, and *Rothia mucilaginosa*, while beneficial bacteria, such as *Parabacteroides distasonis*, *Faecalobacterium prausnitzii* and *Blautia obeum*, exhibit a significant negative correlation with the expression of these three miRNAs. These data could confirm the existence of a relationship between relative abundance of intratumoral breast microbiota, miRNA expression level, and BC metastasis.

This is the first study of BC to integrate intratumoral microbiota profiling and tumoral miRNA expression to predict the development of metastasis using an initial diagnostic tumor sample. Therefore, even if the results are preliminary, the study is novel in its design and innovative in the characterization of metastasis in BC patients.

Finally, we recognize the limitations of our study, such as it being a single-center study, which might impact the generalizability of the results. The sizes of the training and validation cohorts were relatively small for a clinical study. This study only provides preliminary evidence of an association between microbiome, miRNAs, and metastasis in BC; however, these data do not suggest causality. Nevertheless, our study has important strengths, such as the well-phenotyped BC cohort (age balanced) and the use of tumor samples from the time of surgery for the primary tumor, prior to any treatment and the occurrence of metastasis. This approach aims to identify potential markers that may allow us to assess the risk of metastasis development a priori, independent of adjuvant treatment response. Further multicenter studies including a larger number of patients are needed to assess how intratumoral microbial species cross-talk with onco-miRNAs to induce metastasis in BC patients and to validate the clinical utility of the proposed intratumoral microbiota-miRNA signature to detect metastasis in BC patients, potentially leading to more targeted treatment strategies.

## 4. Materials and Methods

### 4.1. Study Patients

A total of 116 patients aged 31–85 years who had undergone primary BC surgery at the Hospital Universitario Virgen de la Victoria (HUVV) in Málaga, Spain, were enrolled in the study between 2006 and 2009. The cohort was divided into two groups: metastatic breast cancer patients (Met-BC, N = 74) and non-metastatic breast cancer patients (NonMet-BC, N = 42). Metastasis occurred between 2 months and 9 years after the initial diagnosis. Patients in the NonMet-BC group did not develop metastasis during the follow-up time of 15 years. Clinicopathological data and follow-up information, encompassing demographic parameters such as age, menopausal status, and tumor characteristics, including tumor grade, diameter, lymph node involvement, Ki-67 index, estrogen receptor (ER)/progesterone receptor (PR) expression, and human epidermal growth factor receptor-2 (Her2) status, and metastasis development, were extracted from pathology reports. Both study groups adhered to the following inclusion criteria: female gender, optimal physical health, absence of any prior history of cancer, pregnancy, or lactation within the preceding 12 months. Exclusion criteria: patients who had received neoadjuvant therapy prior to tumor removal surgery, undefined histological grade in pathological examinations, and non-compliance with study protocols. After tumor removal surgery, the patients received standardized adjuvant treatment and follow-up care: chemotherapy with anthracyclines and taxanes for triple-negative BC, chemotherapy plus anti-HER2 therapy for HER2+ BC, and hormonal therapy (tamoxifen or aromatase inhibitors) for hormone receptor-positive and HER2- BC patients, aligning with international recommendations and scientific evidence [53]. Formalin-fixed and paraffin-embedded (FFPE) breast tumor samples collected at the time of tumor removal surgery before any antitumoral treatment were used for intratumoral microbiota and miRNA expression analysis.

The study protocol was approved by the Medical Ethics Committee at the Virgen de la Victoria University Hospital and was conducted in accordance with the principles of the Declaration of Helsinki. Written informed consent was obtained from all participants.

### 4.2. Immunohistochemistry

The tumor-specific regions in the FFPE BC samples were identified by a pathologist using hematoxylin and eosin (HE) staining. Immunohistochemical staining was performed to determine the intrinsic subtypes of each tumor using specific antibodies for estrogen receptor (ER, clone SP1), progesterone receptor (PR, clone Y85), Ki-67 (clone SP6), epidermal growth factor receptor 1 (EGFR1, clone EP38Y), cytokeratin 5/6 (CK5/6, clone D5/16B4), and HER2 (HercepTestTM, Dako, Denmark). Two pathologists interpreted the immunohistochemical data according to standard protocols in a single-blind manner.

### 4.3. Intratumoral Breast Cancer Microbiota Sequencing

To address the issue of environmental pollution potentially impacting FFPE sample integrity during storage, a method was devised to remove the surface of FFPE blocks and extract internal tissue under sterile conditions within a clean bench environment. Furthermore, rigorous cleaning procedures were implemented for the microtome and blades after processing each patient sample to minimize the risk of cross-contamination. To ensure adherence to the RIDE guidelines, various negative controls, including sampling blank controls, DNA extraction blank controls, and no-template amplification controls, were incorporated into the experimental protocol [54]. DNA extraction from FFPE tumor samples was carried out using the QIAamp DNA FFPE Tissue Kit (Qiagen, Hilden, Germany), following the manufacturer’s guidelines. DNA concentrations were determined using a Qubit 2.0 fluorometer (Invitrogen, Carlsbad, CA, USA). Fifty nanograms of DNA from each FFPE tumor sample was used for the amplification of variable regions (V2, 3, 4, 6–7, 8, and 9) of the 16S rRNA gene using the Ion 16S Metagenomics kit (Thermo Fisher Scientific, Madrid, Spain). Barcoded adapters were ligated to the resultant amplicons and assembled into barcoded libraries using the Ion PlusTM Fragment Library Kit (Thermo Fisher Scientific, Madrid, Spain). These libraries were subsequently combined and templated on an automated Ion Chef system (Thermo Fisher Scientific, Madrid, Spain). Sequencing was performed on an Ion S5 platform (Thermo Fisher Scientific, Madrid, Spain).

### 4.4. Bioinformatics Analysis

The analysis of 16S rRNA amplicons was executed employing QIIME2 (version 2023.7). The q-dada2 plugin, utilizing the DADA2 pipeline, was employed for quality filtering, denoising, dereplication, and chimera filtering of the raw sequence data. The sequence variants obtained through the DADA2 pipeline were consolidated into a unified feature table utilizing the q2-feature-table plugin. All amplicon sequence variants from this merged feature table were clustered into operational taxonomic units (OTUs), employing the open reference clustering method with a 97% sequence similarity cutoff using the q2-vsearch plugin. This clustering was carried out against Greengenes version 13_8, utilizing the OTU reference sequences. Subsequently, the OTUs were aligned utilizing MAFFT (via q2-alignment) and were utilized to construct a phylogenetic tree with fasttree2 (via q2-phylogeny). Taxonomic assignments were made to the OTUs utilizing the q2-feature-classifier classify-sklearn naive Bayes taxonomy classifier. Alpha diversity metrics (Shannon and Chao1), beta diversity metrics (Bray–Curtis dissimilarity and Jaccard indices), and principal coordinate analysis (PCoA) were computed using the q2-diversity plugin after rarefying the samples to a consistent sequencing depth of 999 sequences per sample. The significance of alpha diversity was assessed using the Kruskal–Wallis test, while beta diversity significance was determined using the non-parametric ANOSIM test. The OTU table generated by DADA2 in QIIME 2 was normalized using cumulative sum scaling (CSS) with the R package metagenomeSeq [55].

Metagenome functions were predicted using Phylogenetic Investigation of Communities by Reconstruction of Unobserved States (PICRUSt2) by selecting OTUs paired with the Greengenes database. Statistical analysis was performed in R 3.6.0 using the R heatmap package for analysis and graphical representation. P-values were corrected for multiple comparisons using the Benjamini–Hochberg method (*p* < 0.05).

### 4.5. MiRNAs Identification and Selection

The chosen miRNAs were selected based on a previous study conducted by the research group. In this study, the expression of 1105 miRNAs was analyzed using the Affymetrix miRNA Chip array 2.0 technology (Affymetrix, Santa Clara, CA, USA). The study identified significant differences in the expression of 5 miRNAs (miR-149, miR-10a, miR-20b, miR-30a-3p, and miR-342-5p) between the BC patients with early metastasis regardless of whether it was local, regional, or distant (≤24 months post-surgery) and the non-relapsing group (disease-free at 5 years post-surgery) [19].

### 4.6. RT-qPCR Analysis

RNA was extracted from up to eight 10 µm slides using the RecoverAll Total Nucleic Acid Isolation kit (Invitrogen) following the manufacturer’s instructions. The miRNA concentration was quantified using the specific Qubit microRNA Assay Kit (Invitrogen). Ten nanograms of miRNAs from each FFPE tumor sample was converted to cDNA using the Taqman Advanced miRNA cDNA Synthesis Kit (Applied Biosystems, Waltham, MA, USA). RT-qPCR was performed using Taqman Fast Advanced Master Mix 2X and Taqman Advanced miRNA Assay 20X for six different miRNAs—hsa-miR-149-5p (477917_mir), hsa-miR-10a-5p (479241_mir), hsa-miR-20b-5p (477804_mir), hsa-miR-30a-3p (478273_mir), hsa-miR-342-5p (478044_mir), and hsa-miR-16-5p (477860_mir)—used as reference gene for normalization, with the LightCycler 96 System (Roche, Basel, Switzerland). All assays were conducted in triplicate in accordance with the manufacturer’s guidelines. Relative miRNA expression was calculated using the ΔCt method, with miR-16-5p as the reference gene due to its previously described high stability in breast tissues [19,56,57,58,59].

### 4.7. Statistical Analysis

The Kruskal–Wallis rank-sum test was performed to assess bacterial abundance differences among the study groups. To account for multiple comparisons, we applied the Benjamini–Hochberg method to control the false discovery rate (FDR) for significant *p*-values (*q* < 0.05). The Mann–Whitney U test was used to analyze differences in miRNA expression between the two study groups. The associations between the clinicopathological variables were assessed using Pearson’s chi-square test or Fisher’s exact test.

The Spearman correlation coefficients were calculated to estimate the correlations between the intratumoral bacterial taxa abundance and miRNA expression levels. A linear discriminant analysis (LDA) effect size (LEfSe) algorithm was used for the detection of intratumoral microbiota biomarkers at the species level. The *q*-value was fixed to <0.05, and the threshold used to consider a discriminative feature for the logarithmic LDA score was set to > 3. Random forest (RF) was used to predict the baseline bacteria (species-level relative abundance data) and miRNA expression related to the development of metastasis in BC patients using the training cohort. The default setting of the “randomForest” function carried out in the randomForest R package and a bootstrapping (n = 2000) was used to assess the classification accuracy. Values were considered to be statistically significant when *p* < 0.05. To validate the accuracy of the signature obtained by RF, a validation cohort of 24 BC patients with metastasis and 11 without metastasis was selected from the prospective cohort BC-EPIBIOTA using a randomized algorithm in R study (clinical characteristics of the BC patients in the validation cohort are described in Supplementary File S1). Receiver operating characteristic (ROC) curves and area under the curve (AUC) values were employed to assess the sensitivity of the classifier in both the training and validation cohorts. The Kaplan–Meier curve was used to test the prognostic value of the bacteria and miRNA signature on overall survival in the study patients. All statistical analyses were conducted using SPSS Statistics V.26.0 and GraphPad Prism 9.0 to display the graphical representation. A *p*-value < 0.05 was considered statistically significant.

## 5. Conclusions

This comprehensive study delves into the intricate interplay between miRNA expression, the intratumoral microbiota composition, and their collective impact on metastasis in BC patients. First and foremost, our analysis identified a trio of miRNAs (miR-149-5p, miR-20b-5p, and miR-342-5p), whose expression levels significantly differ between Met-BC and NonMet-BC patients. In parallel, our examination of the intratumoral microbiota unearthed substantial disparities between the two study groups. The Met-BC patients showed heightened richness and diversity at the genus level. Furthermore, beta diversity analyses showed distinct bacterial communities in the Met-BC and NonMet-BC groups, reinforcing the notion that microbiota plays a pivotal role in BC progression. Exploration of the microbiota at finer taxonomic levels revealed significant shifts in the relative abundance of various bacterial genera and species between the two patient groups. The Met-BC group exhibited an increase in several pathogenic and pro-inflammatory species, including *Streptococcus epidermidis*, *Haemophilus influenzae*, *Corynebacterium aurimucosum,* and *Corynebacterium kroppenstedtii*, while the NonMet-BC group displayed higher levels of probiotic bacteria, such as *Parabacteroides distasonis*, *Lactobacillus iners*, *Blautia obeum*, and *Faecalibacterium prausnitzii*. These findings hint at a complex and dynamic relationship between specific bacterial taxa and BC metastasis. Moreover, functional profiling of the microbiota using PICRUSt2 revealed intriguing insights into the potential metabolic pathways associated with metastasis. Notably, bacterial invasion of epithelial cells, transcriptional activity, cell signaling, and extracellular matrix remodeling pathways were enriched in the Met-BC group, underlining their possible roles in BC progression and metastasis. The integration of miRNA expression and species-level bacterial abundance data led to the development of a powerful prognostic signature for metastasis in BC patients in the training and validation cohorts. Finally, the clinical relevance of this signature was underscored by its impact on overall survival. It was found that the low value of this signature was associated with higher overall survival. These results suggest that considering both intratumoral miRNA expression and microbiota abundance in clinical risk assessments of BC could enhance the accuracy of metastasis prediction, potentially leading to more targeted treatment strategies to improve patients’ outcomes in BC.

## Figures and Tables

**Table 1:** Summary of the clinical characteristics of the main cohort of study patients.

		Non-Metastatic BC Patients		Metastatic BC Patients		*p*-Value
		n	(%)	n	(%)	
Number of patients (Total = 116)		42	39.7	74	60.3	
Age at diagnosis	≤50	11	26.2	20	27.0	0.922
	>50	31	73.8	54	73.0	
Hormonal status	Preperim.	12	28.6	19	25.7	0.735
	Postmen.	30	71.4	55	74.3	
Tumor size (cm)	<2	25	59.5	23	31.1	0.009
	2–5	15	35.7	41	55.4	
	>5	2	4.8	10	13.5	
Tumor stage	I	17	40.5	13	17.6	0.007
	II	14	33.3	22	29.7	
	III	11	26.2	39	52.7	
Hystological grade	1	4	9.5	4	5.4	0.237
	2	20	47.6	27	36.5	
	3	17	40.5	42	56.8	
	Unknown	1	2.4	1	1.4	
Histologic subtype	Lobulillar	2	4.8	9	12.2	0.053
	Ductal	34	81.0	61	82.4	
	Medullar	3	7.1	0	0	
	Mixed	1	2.4	4	5.4	
	Papillar	1	2.4	0	0	
	Mucinous	1	2.4	0	0	
	Tubular	1	2.4	0	0	
Intrinsic subtype	Luminal A	7	16.7	16	21.6	0.166
	Luminal B	20	47.6	26	35.1	
	Luminal B-HER2	6	14.3	4	5.4	
	Triple negative	6	14.3	15	20.3	
	HER2-enriched	3	7.1	13	17.6	
Type of surgery	Conservative	34	81	46	62.2	0.036
	Radical	8	19	28	37.8	
Affected lymph node	Negative or unknown	24	57.1	20	27.0	0.001
	1–3	11	26.2	17	23.0	
	≥4	7	16.7	37	50.0	
First-location metastasis	Bone			20	28.4	
	Liver			18	24.3	
	Lymph nodes			5	6.8	
	Skin			4	5.4	
	Pleura			4	5.4	
	Lung			10	13.5	
	Central Nervous System			4	5.4	
	Uterus			1	1.4	
	Ovary			1	1.4	
	Breast			6	8.1	

Preperim.: pre-perimenopausal status; Postmen.: postmenopausal status.

## Data Availability

Data will be made available on request.

## Supplementary Materials

The following supporting information can be downloaded at: https://www.mdpi.com/article/10.3390/ijms25137091/s1.
