# Supplementary material for: Exploring the Relationship between MicroRNAs, Intratumoral Microbiota, and Breast Cancer Progression in Patients with and without Metastasis
Source: Int J Mol Sci. 2024 Jun 28;25(13):7091. doi: 10.3390/ijms25137091 (PMC11241717; doi:10.3390/ijms25137091)
Supplement: Supplementary file 1 [file ijms-25-07091-s001.zip › ijms-3039640-supplementary.pdf]

Supplementary File S1. Clinical characteristic of the breast cancer validation cohort.

		Non-metastatic BC patients		Metastatic BC patients	
		**n**	**(%)**	**n**	**(%)**
**Number of patients (Total= 35)**		11		24	
**Age at diagnosis**	**≤ 50**	2	18.2	6	25.0
	**> 50**	9	81.8	18	75.0
**Hormonal status**	**Preperim.**	2	18.2	6	25.0
	**Postmen.**	9	81.8	18	75.0
**Tumor size (cm)**	**<2**	7	63.6	8	33.3
	**2-5**	4	36.4	14	58.3
	**>5**	0	0	2	8.3
**Tumor stage**	**I**	4	36.4	4	16.7
	**II**	4	36.4	10	41.7
	**III**	3	27.3	10	41.7
**Hystological grade**	**1**	1	9.1	0	0
	**2**	6	54.5	12	50
	**3**	4	36.4	11	45.8
	**Unknown**	0	0	1	4.2
**Histologic subtype**	**Lobulillar**	1	9.1	3	12.5
	**Ductal**	9	81.8	19	79.2
	**Medullar**	1	9.1	0	0
	**Mixed**	0	0	2	8.3
**Intrinsic subtype**	**Luminal A**	3	27.3	3	12.5
	**Luminal B**	5	45.5	11	45.8
	**Luminal B-HER2**	1	9.1	2	8.3
	**Triple negative**	2	18.2	7	29.2
	**HER2-enriched**	0	0	1	4.2
**Type of surgery**	**Conservative**	7	63.6	14	58.3
	**Radical**	4	36.4	10	41.7
**Affected lymph node**	**Negative or unknown**	5	45.5	7	29.2
	**1-3**	4	36.4	6	25.0
	**≥ 4**	2	18.2	11	45.8
**First-location metastasis**	**Bone**			5	20.8
	**Liver**			9	37.5
	**Lymph nodes**			1	4.2
	**Pleura**			1	4.2
	**Lung**			4	16.7
	**Central Nervous System**			2	8.3
	**Breast**			2	8.4

Preperim.: pre-perimenopausal status, Postmen.: postmenopausal status
